# The National Wastewater Surveillance System (NWSS): From inception to widespread coverage, 2020–2022, United States

**DOI:** 10.1016/j.scitotenv.2024.171566

**Published:** 2024-03-09

**Authors:** Carly Adams, Megan Bias, Rory M. Welsh, Jenna Webb, Heather Reese, Stephen Delgado, John Person, Rachel West, Soo Shin, Amy Kirby

**Affiliations:** a Centers for Disease Control and Prevention, 1600 Clifton Road NE, Atlanta, GA 30333, USA; b Epidemic Intelligence Service, Centers for Disease Control and Prevention, 1600 Clifton Road NE, Atlanta, GA 30333, USA

**Keywords:** Wastewater surveillance, Infectious diseases, Public health, COVID-19, SARS-CoV-2, Mpox

## Abstract

Wastewater surveillance is a valuable tool that can be used to track infectious diseases in a community. In September 2020, the Centers for Disease Control and Prevention (CDC) established the National Wastewater Surveillance System (NWSS) to coordinate and build the nation’s capacity to detect and quantify concentrations of SARS-CoV-2 RNA in U.S. wastewater. This is the first surveillance summary of NWSS, covering September 1, 2020 to December 31, 2022. Through partnerships with state, tribal, local, and territorial health departments, NWSS became a national surveillance platform that can be readily expanded and adapted to meet changing public health needs. Beginning with 209 sampling sites in September 2020, NWSS rapidly expanded to >1500 sites by December 2022, covering ≈47 % of the U.S. population. As of December 2022, >152,000 unique wastewater samples have been collected by NWSS partners, primarily from wastewater treatment plants (WWTPs). WWTPs participating in NWSS tend to be larger than the average U.S. WWTP and serve more populated communities. In December 2022, ≈8 % of the nearly 16,000 U.S. WWTPs were participating in NWSS. NWSS partners used a variety of methods for sampling and testing wastewater samples; however, progress is being made to standardize these methods. In July 2021, NWSS partners started submitting SARS-CoV-2 genome sequencing data to NWSS. In October 2022, NWSS expanded to monkeypox virus testing, with plans to include additional infectious disease targets in the future. Through the rapid implementation and expansion of NWSS, important lessons have been learned. Wastewater surveillance programs should consider both surge and long-term capacities when developing an implementation plan, and early standardization of sampling and testing methods is important to facilitate data comparisons across sites. NWSS has proven to be a flexible and sustainable surveillance system that will continue to be a useful complement to case-based surveillance for guiding public health action.

## Graphical Abstract

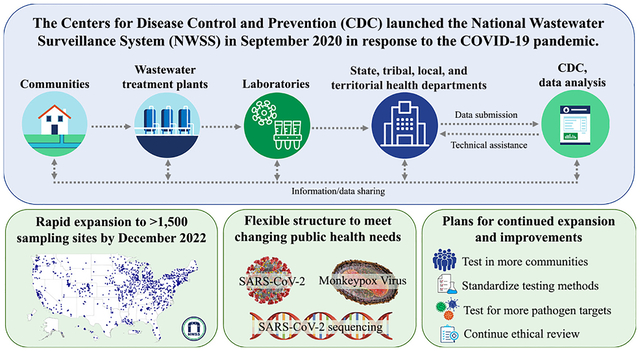

## 1. Introduction

Wastewater surveillance is a valuable tool that can be used to track infectious diseases and other markers of public health importance in a community (Kilaru et al., 2022). Some pathogens are shed by infected individuals in ways that end up in wastewater (e.g., stool, urine, oral/nasal secretions, and sloughing of skin); thus, their detection in wastewater can be used to inform public health decisions. Wastewater surveillance involves collecting a sample of untreated wastewater from a sewershed (the community area served by a wastewater collection system), often as it flows into a wastewater treatment plant (WWTP), and running analyses to detect and quantify concentrations of microbial or chemical targets (Sims and Kasprzyk-Hordern, 2020). WWTPs have clearly defined sewersheds, so residents of a sewershed (i.e., the community that primarily contributes to a sample) can be readily determined. Thus, wastewater provides a convenient, pooled sample of individuals connected to a sewer network. Unlike clinical surveillance data, wastewater surveillance data are independent of clinical symptoms, healthcare-seeking behavior, and testing access (Peccia et al., 2020). Therefore, wastewater surveillance data can be used to further understand levels of infection in a community, especially when infected individuals may be asymptomatic or unlikely to seek healthcare or testing. Moreover, wastewater data can be available within days of viral shedding onset and can provide an early warning that levels of infection are increasing. Taken together, wastewater surveillance can be a useful complement to clinical surveillance for tracking levels of infection in a community.

Although wastewater surveillance has been used for decades to monitor global disease trends, it expanded dramatically during the COVID-19 pandemic (Kilaru et al., 2022). In the United States, wastewater surveillance was not used widely nor routinely before the pandemic. In response to the pandemic, the Centers for Disease Control and Prevention (CDC) launched the National Wastewater Surveillance System (NWSS) in September 2020 to coordinate and build the nation’s capacity to detect and quantify the presence of SARS-CoV-2 RNA in U.S. wastewater (Kirby et al., 2021). By partnering with state, tribal, local, and territorial health departments (STLTs), NWSS transformed independent, local wastewater surveillance efforts into a robust national surveillance system. This paper provides the first surveillance summary of NWSS, from its launch in 2020 through December 31, 2022. The purpose of this paper is to provide an informational resource on the surveillance system for NWSS partners, other governmental organizations, non-governmental organizations, and the general public. To this end, this paper describes NWSS’s structure and design and summarizes NWSS sampling sites and sampling methods. It also summarizes the utility and limitations of NWSS data, lessons learned during NWSS implementation, and future directions of the surveillance system.

## 2. Materials and methods

We retrospectively summarized sample-level NWSS wastewater surveillance data for the period September 1, 2020 to December 31, 2022. The purpose was to provide annual summary statistics for sampling site characteristics, sample characteristics, and populations served by NWSS sites. Summary statistics for December 2022 were also provided to summarize data from sites actively collecting samples at the end of the surveillance summary period. Sampling site population coverage was compared to the U.S. population using the following datasets: 1) U.S. Environmental Protection Agency (EPA) Enforcement and Compliance History Online (ECHO) Integrated Compliance Information System (ICIS) (National Enforcement and Compliance History Online Data Downloads, 2023), 2) EPA Facility Registry Service (FRS) (Geospatial Data Download Service, n.d.), 3) U.S. Census Bureau 2020 Decennial Census Data (Decennial Census, 2021), 4) U.S. Department of Agriculture 2013 Rural-Urban Continuum Codes (Rural-Urban Continuum Codes, 2013), and 5) CDC 2020 Social Vulnerability Indices (CDC/ATSDR Social Vulnerability Index, 2023). Descriptive statistics were generated using R software (version 4.3.1, R Foundation). NWSS recommendations are intended for NWSS partners and were current at the time of publication; recommendations may change over time. This activity was reviewed by CDC and was conducted consistent with applicable federal law and CDC policy.^1^

## 3. NWSS implementation, structure, and design

The initial goal of NWSS was to rapidly increase wastewater surveillance coverage in the United States in response to the COVID-19 pandemic. To this end, CDC partnered with state, territorial, and local health departments, who enrolled sampling sites (i.e., sites) into NWSS and identified laboratories to conduct wastewater testing. Although site selection priorities varied by health department, common goals were to increase wastewater surveillance geographic and population coverage. Some health departments also prioritized enrolling sites in areas with high social vulnerability indices (SVI) (CDC/ATSDR Social Vulnerability Index, 2023), low COVID-19 vaccination rates, increased occupational risks (e.g., areas with meat and poultry processing facilities), and/or high COVID-19 rates. As of December 2022, NWSS continues to expand its geographic and population coverage in the United States and U.S. territories.

NWSS provides funding to state, territorial, and local health departments for wastewater surveillance activities through CDC’s Epidemiology and Laboratory Capacity for Prevention and Control of Emerging Infectious Diseases Cooperative Agreement (ELC), a national funding strategy to support state, territorial, and local capacities for emerging infectious disease control. To facilitate the rapid expansion of surveillance coverage, NWSS also awarded two commercial contracts—one from November to April 2022 and one from April 2022 to January 2023. Sites were selected for enrollment into the NWSS commercial contracts based on communications with STLT health departments and standard selection criteria, including SVI, rural-urban continuum codes (RUCC) (Rural-Urban Continuum Codes, 2013), and COVID-19 vaccination rates. Importantly, commercial contracts facilitated enrollment of sites on tribal lands, after consultation with tribal leaders and health departments. In April 2022, sites from the first commercial contract were transferred to the second, after which additional sites were enrolled into the second commercial contract. All wastewater samples from the second commercial contract were tested at one laboratory, and the contractor compiled and reported the data to CDC. The contractor also sent all residual wastewater samples to the CDC Biorepository for storage and potential future testing by CDC.

In October 2022, NWSS also began receiving wastewater data from an academic partner program, including historic data from samples collected as early as January 2022. In the future, NWSS may also receive data from additional sources, as long as those data meet NWSS’s data quality standards. For data to meet NWSS’s data quality standards, they must include sample-level information, quantitative concentration results for a target’s genetic material (e.g., SARS-CoV-2 RNA) in wastewater, and all required variables (Wastewater Surveillance Data Reporting and Analytics, 2023).

## 4. Wastewater sampling sites

**Fig. 1.**
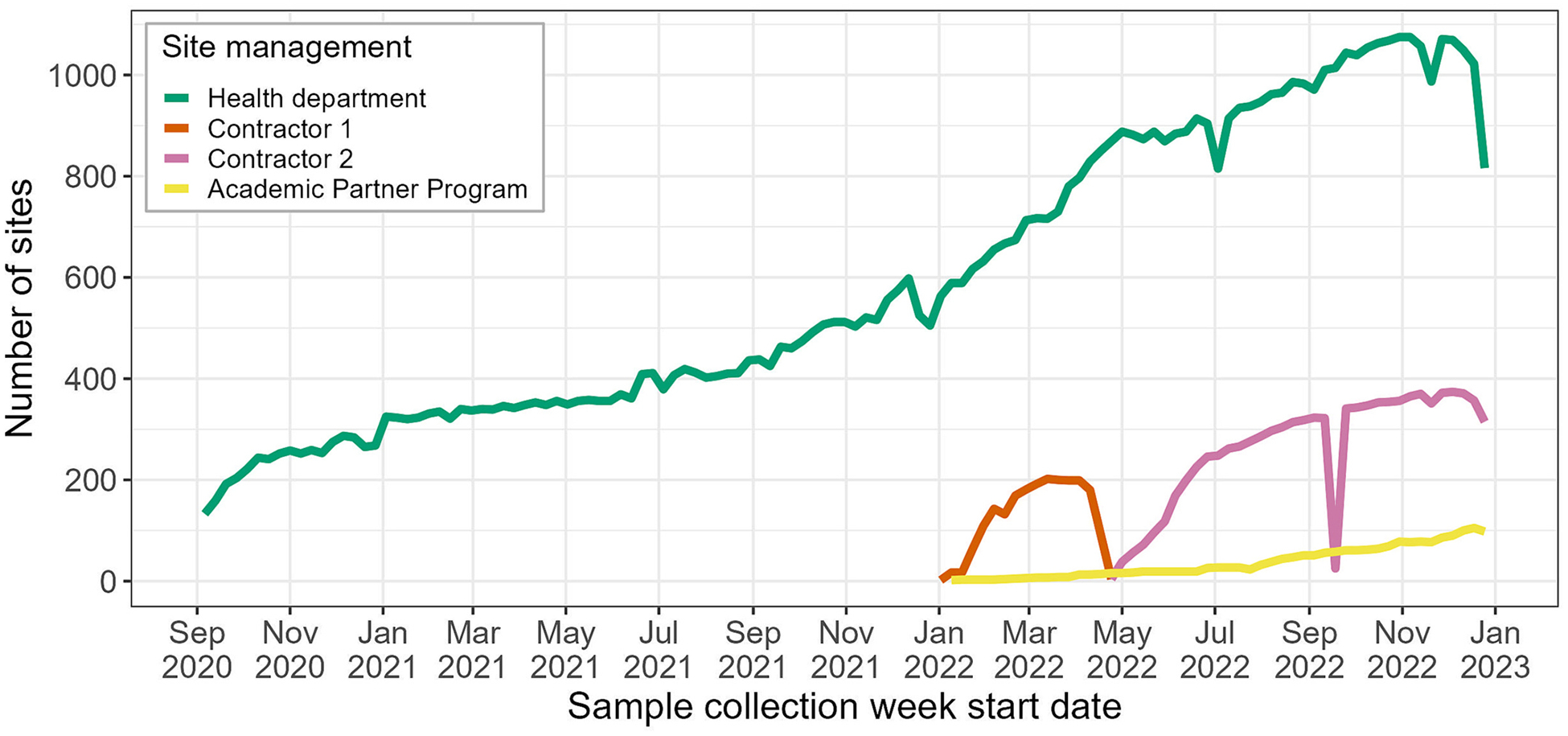
Number of wastewater surveillance sampling sites participating in the CDC’s National Wastewater Surveillance System (NWSS) by week^a,b^: September 1, 2020 – December 31, 2022^c^. Abbreviations: CDC, Centers for Disease Control and Prevention. ^a^ Sites that collected ≥1 sample in a week (Sunday-Saturday) were included in that week’s counts. ^b^ The decline in health department sites collecting samples at the end of December 2022 was most likely due to reduced sampling over the holiday period. The number of health department sites sampling in January 2023 returned to pre-holiday levels (not shown). ^c^ Data were downloaded August 31, 2023 and restricted to samples collected prior to January 1, 2023.

**Fig. 2.**
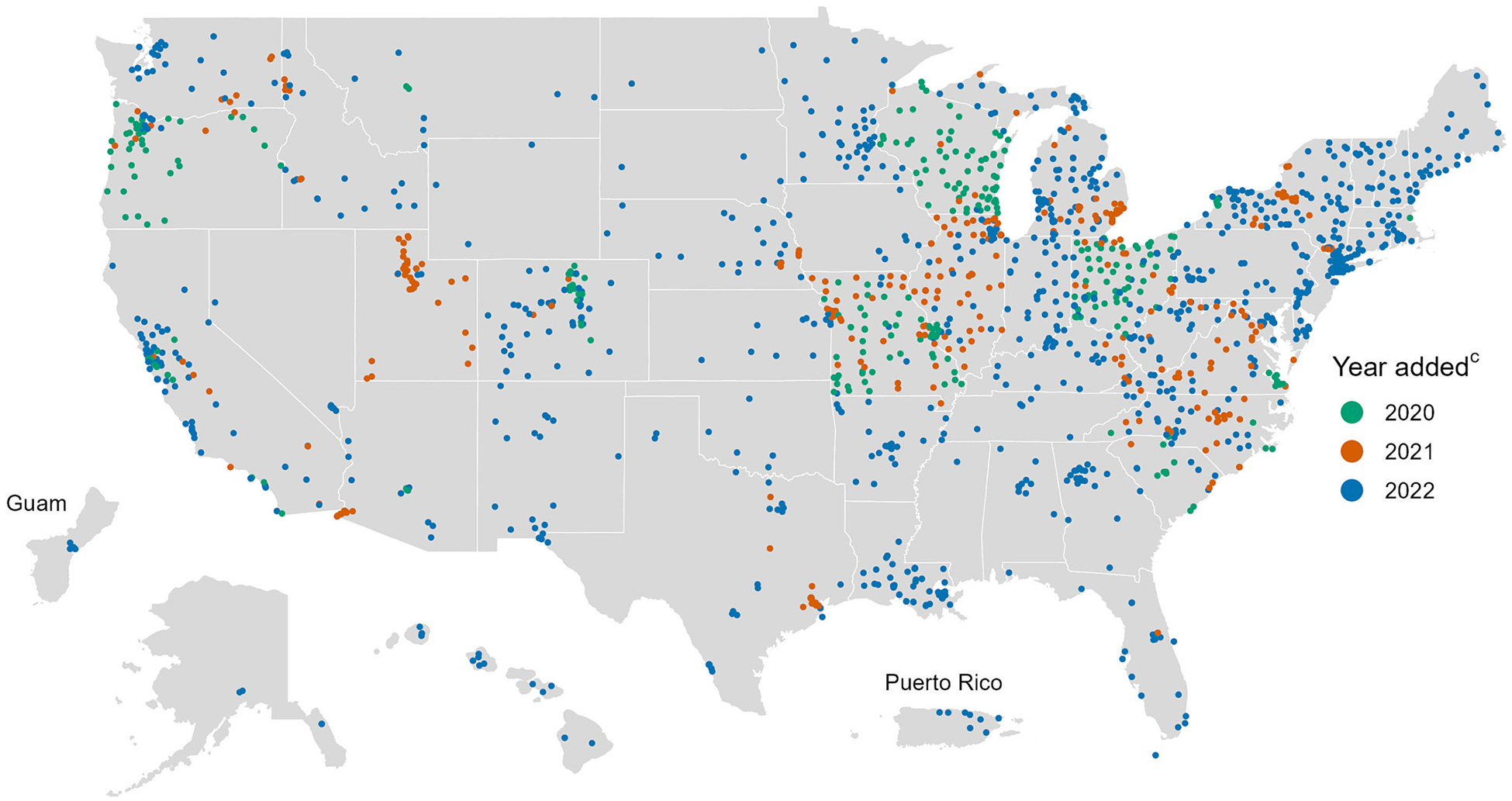
Locations of wastewater surveillance sites^a,b^ participating in the CDC’s National Wastewater Surveillance System (NWSS): September 1, 2020 – December 31, 2022. Abbreviations: CDC, Centers for Disease Control and Prevention. ^a^ Each point on the map represents a wastewater surveillance site. A site can represent all or part of a sewershed, which is the geographic area contributing wastewater to a sampling location. Sewersheds may cross county or state boundaries. ^b^ Sites are plotted at the centroid of the ZIP code in which they are located and then jittered, so points on the map do not correspond to exact sampling locations. ^c^ Year added was determined by the first sample collection date for a site.

When NWSS was established in September 2020, there were initially 209 sampling sites. This increased to 293 sites by December 2020, 621 sites by December 2021, and 1567 sites by December 2022 (Table 1, Figs. 1–2). The proportion of the U.S. population served by NWSS sites similarly increased, from 12 % in 2020 to 45 % in 2022 (Table 1). In total, 1799 sampling sites participated in NWSS between September 1, 2020 and December 31, 2022, serving nearly 155 million people, or ≈47 % of the U.S. population and ≈58 % of the U.S. population connected to a municipal sewage system. Although most sampling sites continue to participate in NWSS after they enroll, sites may choose to stop collecting samples for various reasons, including a lack of resources or support for wastewater surveillance activities. Therefore, the number of sites acitvely collecting samples at year’s end is slightly less than the number of sites collecting samples throughout the year (Table 1).

Initially, the majority of NWSS sites were located in U.S. Health and Human Service (HHS) Regions 5 and 7, but NWSS has since expanded and become more geographically representative of the United States (Table 2, Fig. 2). As of December 2022, sites are located in all 50 states, five major cities, two U.S. territories, and six tribal lands. Most of these sites (71 %) are managed by state or local health departments, whereas 22 % are managed by NWSS’s commercial contractor and 6 % are managed by an academic partner program.

Sites can be located at a WWTP or upstream of a WWTP, such as a pump station, manhole, healthcare facility, correctional facility, university, or school (Table 1). The majority (89 %) of all NWSS sites have been located at WWTPs. As of December 2022, nearly 1400 WWTPs are actively collecting samples for NWSS (i.e., collected at least one sample in December 2022), representing ≈8 % of all WWTPs in the United States. Compared to all U.S. WWTPs, WWTPs participating in NWSS are substantially larger, receiving a median of 6.1 (IQR: 2.2, 18.2) millions of gallons of wastewater per day (MGD), compared to a median of 0.4 (IQR: 0.1, 1.8) MGD. This indicates that STLT health departments and CDC have prioritized enrolling WWTPs that serve larger populations.

WWTPs can collect wastewater that includes both sewage and stormwater (i.e., a combined sewer system), or sewage only. From 2020 to 2022, the proportion of WWTPs participating in NWSS collecting wastewater from a combined sewer system increased from 26 % to 44 % (Table 1). This has implications for data interpretation, as unadjusted wastewater concentrations of SARS-CoV-2 may fluctuate more when collected from a combined sewer system, depending on precipitation levels. Furthermore, not all wastewater collected by WWTPs is from household or building (e.g., schools, offices, healthcare facilities) use. From 2020 to 2022, approximately 5 % of wastewater received by WWTPs participating in NWSS was from industrial sources (e.g., manufacturing and power generation).

**Fig. 3.**
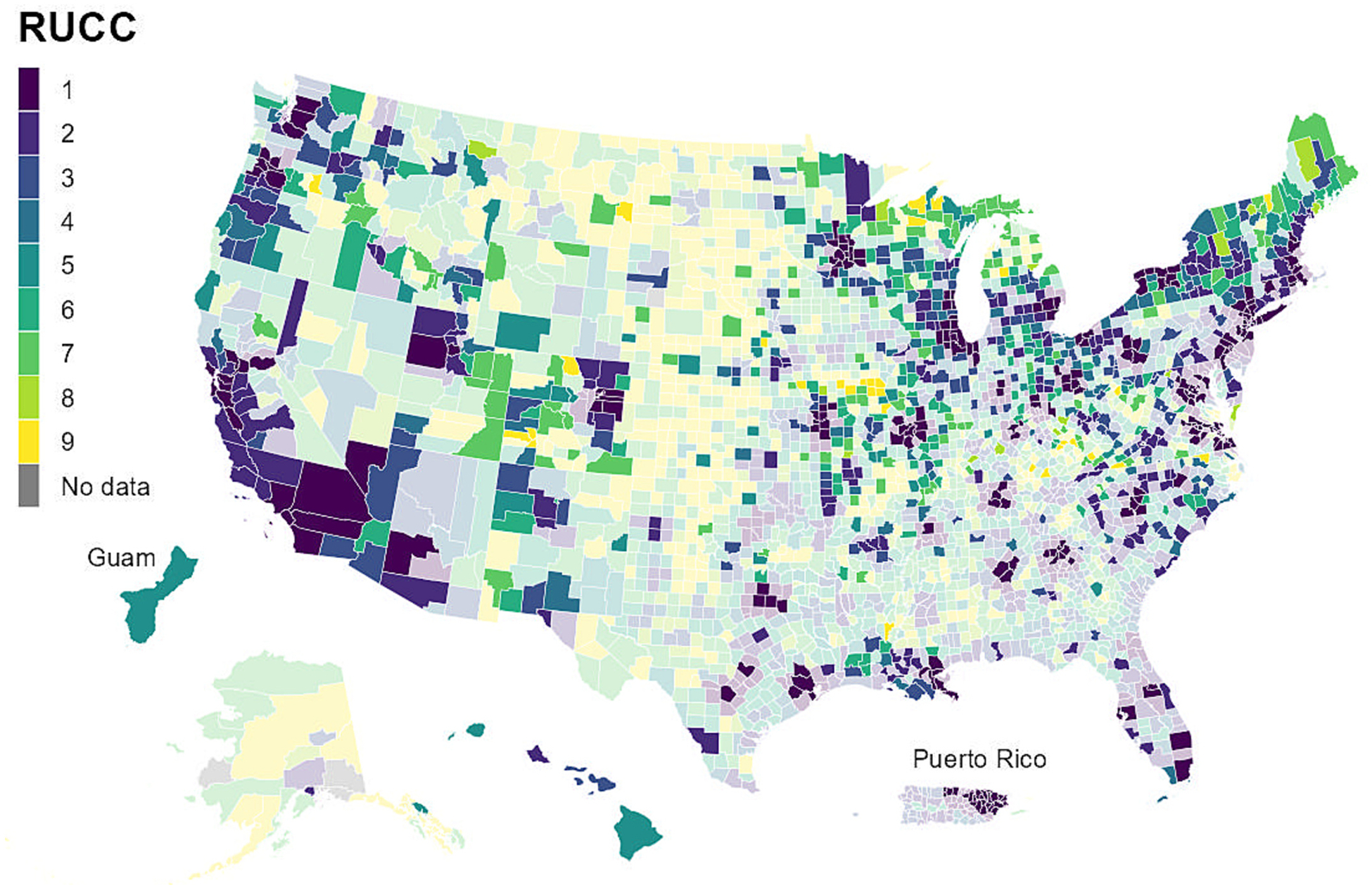
Counties in the United States and U.S. territories^a^ by Rural-Urban Continuum Codes (RUCC)^b^; counties covered by CDC’s National Wastewater Surveillance System (NWSS)^c^ are shown by brighter colors: September 1, 2020 – December 31, 2022. Abbreviations: CDC, Centers for Disease Control and Prevention; RUCC, Rural-Urban Continuum Codes. ^a^ U.S. territories include Guam and Puerto Rico. ^b^ Data are from the U.S. Department of Agriculture (USDA) 2013 Urban-Rural Continuum Codes dataset: https://www.ers.usda.gov/data-products/rural-urban-continuum-codes/. RUCC is a classification from 0 to 9, with higher values indicating more rural counties. ^c^ Counties are considered to be covered by NWSS if they were served entirely or partially by one or more NWSS sampling site (i.e., at least 1 site serving the county collected at least 1 sample between September 1, 2020 and December 31, 2022). A site can serve all or part of a sewershed, which is the geographic area contributing wastewater to a sampling location. Sewersheds may cross county or state boundaries.

**Fig. 4.**
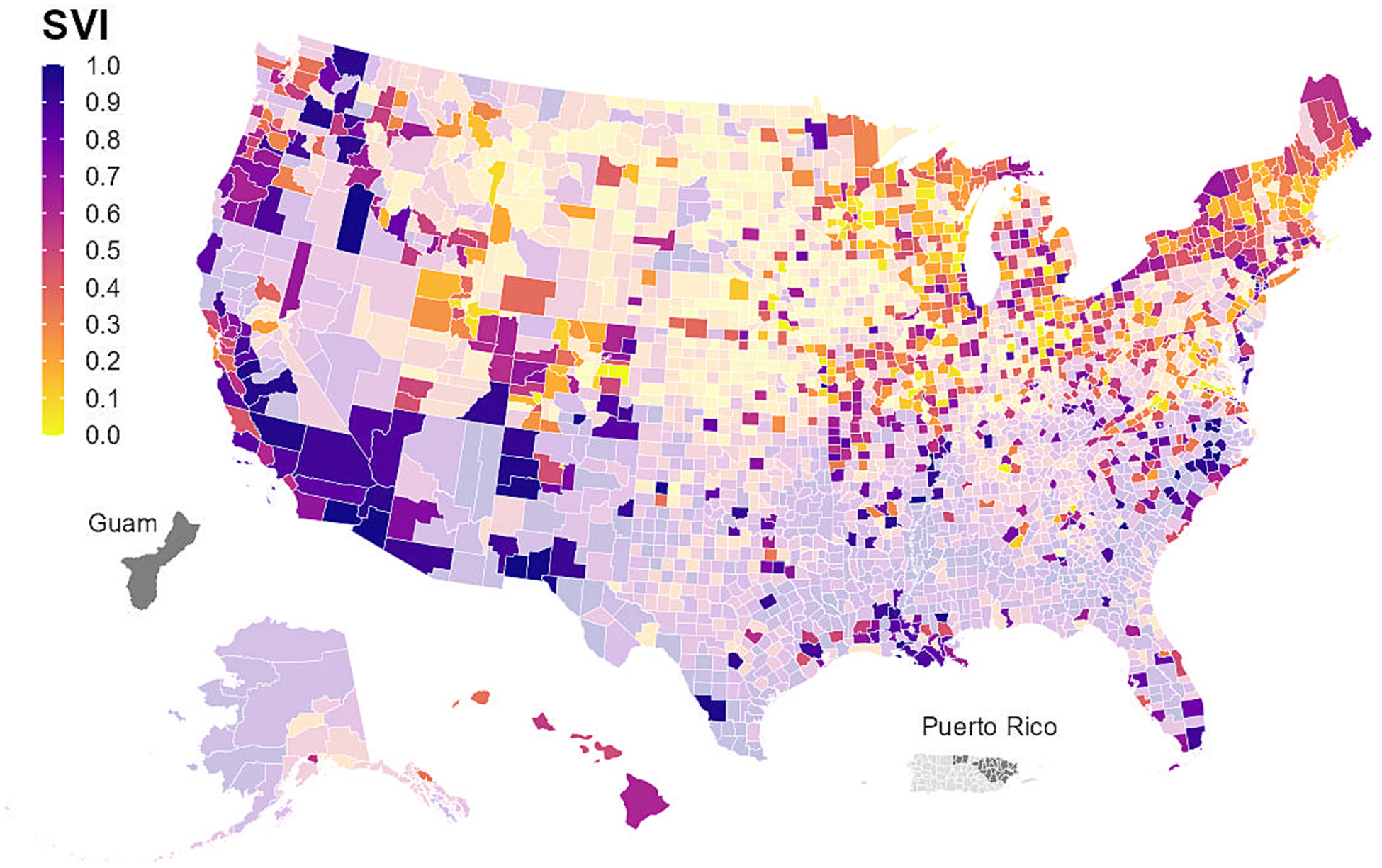
Counties in the United States and U.S. territories^a^ by Social Vulnerability Index (SVI)^b^; counties covered by CDC’s National Wastewater Surveillance System (NWSS)^c^ are shown by brighter colors: September 1, 2020 – December 31, 2022^d.^ Abbreviations: CDC, Centers for Disease Control and Prevention; SVI, Social Vulnerability Index. ^a^ U.S. territories include Guam and Puerto Rico. ^b^ Data are from the CDC/Agency for Toxic Substances and Disease Registry (ATSDR) 2020 Social Vulnerability Index dataset: https://www.atsdr.cdc.gov/placeandhealth/svi/index.html. SVI is a measure from 0 to 1, with higher values indicating greater vulnerability in a public health emergency. ^c^ Counties are considered to be covered by NWSS if they were served entirely or partially by one or more NWSS sampling site (i.e., at least 1 site serving the county collected at least 1 sample between September 1, 2020 and December 31, 2022). A site can serve all or part of a sewershed, which is the geographic area contributing wastewater to a sampling location. Sewersheds may cross county or state boundaries. ^d^ Counties with missing SVI data are shown in gray.

NWSS sampling sites can serve all or part of a sewershed, which typically do not follow any administrative boundaries (e.g., county boundaries). Between 2020 and 2022, a total of 997 U.S. counties were served, at least in part, by one or more NWSS sampling site, representing 31 % of all U.S. counties. The number of counties served by one or more NWSS site more than quadrupled, from 211 counties in 2020 to 976 counties in 2022 (Table 2). Counties served by NWSS sites are more likely to include urban areas with larger populations compared to all U. S. counties (Table 2, Fig. 3). However, NWSS sites are increasingly expanding to less populated counties (Table 2). Additionally, NWSS sites serve counties that have slightly lower SVI than all U.S. counties, with lower values indicating less vulnerability in a public health emergency (Table 2, Fig. 4). At the time of publication, several health departments and NWSS’s commercial contractor were prioritizing the enrollment of sampling sites in rural communities and communities with high SVI, as wastewater surveillance may improve disease burden estimates in these communities and help promote health equity.

## 5. Wastewater sampling methods

Depending on the site, NWSS samples may be collected by WWTPs, laboratories, health departments, or environmental agencies. Between September 2020 and December 2022, NWSS received data from >153,000 unique wastewater samples collected by NWSS partners. There are two main types of samples: untreated wastewater and primary sludge (Developing a Wastewater Surveillance Sampling Strategy, 2023; SARS-CoV-2 Wastewater Surveillance Testing Guide for Public Health Laboratories, 2022). Untreated wastewater consists of sewage only or both sewage and stormwater; it can be collected from a WWTP or upstream. Primary sludge consists of suspended solids that settle out of wastewater during the first solids removal process at a WWTP; it can only be collected from a WWTP. Primary sludge may have more concentrated levels of SARS-CoV-2, but it can be more challenging to collect and is not available at all WWTPs (Aghababaei et al., 2023). The majority (79 %) of NWSS samples collected in December 2022 were untreated wastewater (Table 1).

There are two main methods that can be used to collect samples: grab sampling and composite sampling (Developing a Wastewater Surveillance Sampling Strategy, 2023; SARS-CoV-2 Wastewater Surveillance Testing Guide for Public Health Laboratories, 2022). Grab samples are from a single moment in time; they are highly influenced by daily fluctuations in wastewater flow and composition. Composite samples are multiple grab samples pooled at a specified frequency over a set period of time (typically 24 h). They are considered more representative of community contributions to wastewater than grab samples but usually require automated equipment to obtain (SARS-CoV-2 Wastewater Surveillance Testing Guide for Public Health Laboratories, 2022). Composite samples can be time-weighted (collected at preset time intervals) or flow-weighted (collected over time intervals with volume proportional to the flow rate), but flow-weighted sampling typically requires more resources (Wastewater Surveillance Common Terms to Know, 2023). NWSS recommends that sampling sites use time-weighted composite sampling when feasible. Nearly half of all NWSS samples collected in December 2022 were time-weighted composite (Table 1).

The frequency of sample collection varies by site, but all sites are encouraged to sample at least twice weekly (Feng et al., 2021). In December 2022, sites collected a median of 2 (IQR: 1, 2) samples per week (Table 1).

## 6. Wastewater testing methods

NWSS sites send samples for testing to partner laboratories, including public health, environmental, academic, and commercial laboratories. NWSS recommends that laboratories refrigerate samples at 4 °C immediately after collection and, if possible, process samples on the day of receipt. If needed, laboratories may store samples at 4 °C for up to four days before processing (SARS-CoV-2 Wastewater Surveillance Testing Guide for Public Health Laboratories, 2022). Since 2020, the median time from sample collection to testing has consistently been 2 days (IQR: 1, 4) (Table 1). Between 2020 and 2022, 112 laboratories participated in NWSS, with 78 laboratories actively testing samples in December 2022.

Laboratories use different methods for testing wastewater samples, depending on laboratory capacity and local and national public health data needs. There are four main wastewater laboratory testing steps: 1) sample preparation, 2) sample concentration, 3) extraction of viral genetic material, and 4) viral quantification. For viral quantification, NWSS recommends that laboratories use reverse transcription digital polymerase chain reaction (RT-dPCR) or reverse transcription droplet digital polymerase chain reaction (RT-ddPCR), as opposed to reverse transcription quantitative polymerase chain reaction (RT-qPCR), because these platforms are less subject to inhibition (SARS-CoV-2 Wastewater Surveillance Testing Guide for Public Health Laboratories, 2022). In 2022, most samples (55 %) were tested using either RT-dPCR (or dPCR) or RT-ddPCR (or ddPCR), which was an increase from 2020 (42 %) and 2021 (45 %) (Table 1). To ensure the quality of results, NWSS recommends that laboratories perform triplicate PCR reactions for each sample. If the relative standard deviation across triplicates is >20 %, NWSS recommends that samples be re-run (SARS-CoV-2 Wastewater Surveillance Testing Guide for Public Health Laboratories, 2022).

There are five laboratory quality control checks that NWSS recommends (SARS-CoV-2 Wastewater Surveillance Testing Guide for Public Health Laboratories, 2022; Wastewater Surveillance Testing Methods, 2023). First, a matrix recovery control (e.g., bovine coronavirus) should be added to samples prior to pasteurization to estimate the amount of viral loss during sample processing. Second, a human fecal normalization control should be measured in samples to estimate its human fecal content. Concentrations of SARS-CoV-2 can then be normalized using human fecal controls to account for variability in human fecal content and viral loss from source to test (SARS-CoV-2 Wastewater Surveillance Testing Guide for Public Health Laboratories, 2022). In December 2022, the majority (66 %) of samples were tested for pepper mild mottle virus as a human fecal normalization control. Third, a quantitative positive control should be added to samples to measure the amount of RNA loss throughout the entire process. Fourth, during initial method validation or testing from a new location, inhibition assessments should be performed to evaluate and quantify the extent of molecular amplification inhibition. Although inhibition evaluation may be performed more regularly, PCR inhibition has been infrequently observed in wastewater surveillance testing, suggesting that routine inhibition evaluation may be unnecessary (SARS-CoV-2 Wastewater Surveillance Testing Guide for Public Health Laboratories, 2022). Finally, negative controls (e.g., method blank, extraction blank, and no template controls) should be processed in parallel with all wastewater samples to test for contamination during the laboratory workflow. If any amplification is observed in the negative controls, NWSS recommends that samples be retested and that all reagents in the extraction kit/protocol be discarded.

## 7. Data submission, processing, and display

Once laboratory testing is complete, laboratories submit SARS-CoV-2 concentration data to health departments, the commercial contractor, or the academic partner program (collectively referred to as partners). Partners submit wastewater concentration data to CDC using a standardized comma separated value (CSV) file. Data are submitted through the Data Collation and Integration for Public Health Event Response (DCIPHER), a cloud-based data integration and management platform. In addition to wastewater concentration data, partners also submit sewershed boundary polygon data and laboratory protocols.

Partners report average concentrations for triplicate PCR reactions to NWSS. As of December 31, 2022, there are 86 variables in the NWSS dataset, 39 of which are required for data submission. NWSS recommends that partners upload data to DCIPHER at least once weekly. Once upload is complete, the data file is pushed through an automated DCIPHER pipeline that cleans, parses, and analyzes the data. The analyzed data contain four metrics of SARS-CoV-2 wastewater concentration trends (Table 3). Health departments can view the analyzed data on the DCIPHER dashboard and the public can view a subset of the analyzed data on the CDC COVID Data Tracker webpage (COVID Data Tracker, 2023).

## 8. SARS-CoV-2 variant tracking

Wastewater surveillance genomics can be used to track variants of a pathogen over time and to detect the emergence of novel variants in a community (COVID Data Tracker, 2023). Sequencing wastewater from a community typically involves sequencing genetically diverse, mixed populations of multiple, separate cocirculating genomes. To analyze these data, bioinformatic pipelines that are specific to variant detection in wastewater samples are needed (SARS-CoV-2 Wastewater Surveillance Testing Guide for Public Health Laboratories, 2022). Although bioinformatic pipelines built for clinical samples also exist, using these pipelines for wastewater samples will often result in a consensus genome assembly that does not represent any true genomes present in the sample. Rather, the consensus genome assembly may be a chimera or artifact sequence formed by two or more biological sequences incorrectly joined together. To address this issue, bioinformatic pipelines built for wastewater samples do not determine a single, consensus genome, but rather estimate the proportion of different variants that comprise each sample.

NWSS began receiving SARS-CoV-2 sequencing data from partners in July 2021. To initiate sequencing, NWSS provided a data dictionary to the U.S. Food and Drug Administration (FDA) that was used to develop a step-by-step protocol for health department partners to submit SARS-CoV-2 sequencing data to the National Center for Biotechnology Information (NCBI) Sequence Read Archive (SRA) database. The NCBI SRA is a public database that facilitates global coordination of genomic disease surveillance data (SARS-CoV-2 Wastewater Surveillance Testing Guide for Public Health Laboratories, 2022). Health department partners, along with NWSS’s commercial contractor, submit wastewater sequencing data and its contextual metadata to the NCBI SRA, where it can be accessed for general public health uses. These data are also pulled into DCIPHER, where NWSS analyzes and visualizes the data. As of December 31, 2022, approximately 27,000 NWSS samples from all 50 states, two territories, two cities, and three tribes have been sequenced. Sequencing data from these samples are publicly available.

NWSS determines the dominant variant for a given site based on the highest relative lineage abundance from mixed SARS-CoV-2 samples by site for a given day. Different quality control thresholds can be selected for the calculations. The dominant variant(s) from a site’s most recent sequenced sample are displayed to NWSS partners on the DCIPHER dashboard and to the public on COVID Data Tracker.

## 9. Monkeypox virus testing

In response to the global 2022 mpox outbreak, which began in May 2022 and resulted in the unprecedented spread of mpox outside endemic countries, NWSS expanded to include monkeypox virus (MPXV) testing. NWSS collaborated with its commercial contractor in September 2022 to develop laboratory methods for testing wastewater for MPXV (Acer et al., 2022). By October 2022, NWSS’s commercial contractor had started routinely testing wastewater for MPXV.

Leveraging existing sites, NWSS’s commercial contractor added MPXV testing in a phased approach, beginning with 28 sites in October 2022 and expanding to 376 sites by December 31, 2022. Because samples were tested for both SARS-CoV-2 and MPXV, no additional sampling was required. In October 2022, NWSS also began receiving MPXV wastewater data from an academic partner program, which had begun testing wastewater for MPXV in June 2022 (Wolfe et al., 2023; Boehm et al., 2023). As of December 2022, a total of 460 sites (managed by the commercial contractor or academic partner program) are testing wastewater for MPXV (Table 1). Sites are located in 48 states and two U. S. territories. By December 2022, a total of 6831 samples have been tested for MPXV, of which 573 (8 %) from 140 (30 %) sites have tested positive.

Due to NWSS’s flexible structure, it was a simple process to add MPXV to data submissions and displays. NWSS partners submit MPXV data to CDC through DCIPHER using the same CSV file used to submit SARS-CoV-2 data. MPXV wastewater data became viewable to health departments on the DCIPHER dashboard in December 2022 and a subset of the MPXV data became viewable to the public on a CDC mpox webpage in February 2023 (U.S. Mpox Wastewater Data, 2023).

### 9.1. NWSS utility

Health department partners have used NWSS data to support and guide public health actions to mitigate COVID-19. As of December 2022, STLT health departments have used NWSS data to: 1) independently support increases or decreases in cases (Kirby et al., 2021), 2) provide an early warning for increases in infections (Kirby et al., 2021), 3) inform public health messaging (Hopkins et al., 2023; Valencia et al., 2023), 4) complement case-based surveillance data in communities with limited clinical testing (Kirby et al., 2021), 5) inform the public of wastewater trends through jurisdiction-specific, public-facing dashboards (Hopkins et al., 2023; Valencia et al., 2023), 6) forecast cases or hospital utilization (Hopkins et al., 2023; Hill et al., 2023), 7) inform targeted clinical testing and vaccination efforts (Kirby et al., 2021; Hopkins et al., 2023; Valencia et al., 2023), 8) examine the impact of home testing on case reporting (Varkila et al., 2023), and 9) detect the emergence of SARS-CoV-2 variants (Valencia et al., 2023; Kirby et al., 2022; Lambrou et al., 2023). Furthermore, although NWSS does not currently test for poliovirus, health departments have leveraged NWSS funding and resources to enable poliovirus testing in select communities.

### 9.2. NWSS limitations

There are several limitations to consider when using NWSS data. First, although WWTPs have defined sewersheds, obtaining geographic data that delineate sewershed boundaries can be challenging. Therefore, as of December 31, 2022, geographic information on sewershed boundaries is only available for about a quarter of NWSS sites, making it difficult to accurately assess population coverage. Second, sampling frequency and methods used to collect and test wastewater samples vary by site, which makes it challenging to compare SARS-CoV-2 wastewater levels across sites and time. This also makes it challenging to aggregate data to provide trend estimates for states, regions, and the nation. Third, approximately 20 % of U.S. households do not connect to municipal sewage systems and therefore may not be represented by NWSS data (unless individuals in those households visit other places, such as work or school, that are connected to municipal sewage systems) (About Septic Systems. United States Environmental Protection Agency, 2023). This includes homes on septic systems and communities or facilities served by decentralized wastewater treatment facilities (About Small Wastewater Systems, 2023). Moreover, individuals who do not use a toilet (e.g., people who wear diapers) are also not captured through wastewater surveillance. Fifth, the limit of detection (i.e., the minimum number of infected individuals who must be present for wastewater surveillance to detect the pathogen) for SARS-CoV-2 and other pathogens has yet to be established, meaning an absence of detection may not indicate an absence of infections in a community. However, for SARS-CoV-2, we can estimate the probability that a community is free of infections given absences of detection over time (Larsen et al., 2022). Lastly, wastewater data at this time cannot be used reliably and accurately to predict the number of individuals infected with SARS-CoV-2 (Soller et al., 2022; McMahan et al., 2021). After considering these limitations, wastewater surveillance should not replace other surveillance systems, but can be a useful complement to help guide public health action.

### 9.3. Lessons learned

NWSS transformed independent, local wastewater surveillance efforts into a unified national surveillance system. Through this process, CDC’s NWSS team learned valuable lessons about rapidly implementing and expanding a national surveillance program during a pandemic response. These lessons learned are intended to provide information on how state and local wastewater surveillance programs, as well as other countries’ national wastewater surveillance programs, can be successful.

First, wastewater surveillance programs should consider both surge and long-term capacities when developing an implementation plan. NWSS was implemented rapidly in response to the COVID-19 pandemic. As such, the focus was initially on pandemic response. However, building long-term capacity, although time-intensive and sometimes not compatible with outbreak or pandemic response needs, is necessary for building a sustainable program. NWSS is working to build a long-term surveillance platform through partnerships with STLT health departments. To account for surge capacity, NWSS has supplemented its surveillance platform with a commercial contract that can be rapidly deployed (e.g., for the 2022 mpox outbreak response) and a partnership with the U.S. Geologic Survey that provides rapid testing support (Holst et al., 2022). As NWSS continues to be a valuable complement to case-based surveillance for pandemic and outbreak response, ensuring its long-term capacity is a priority.

Second, wastewater surveillance programs should intentionally select sampling locations to meet sampling objectives. Although sampling sites are selected by health departments, with support from CDC, site selection should support NWSS program-level surveillance objectives for SARS-CoV-2: 1) provide early detection of changes in epidemiological patterns, 2) assess trends in circulation, and 3) track circulating variants. Health departments may have additional surveillance objectives, dependent on local priorities and public health needs. Although feasibility must be considered (e.g., available resources and WWTP willingness and ability to participate), a conscious effort should be made to select sampling locations that meet the sampling objectives. This can lead to improvements in public health situational awareness and advancement of health equity by expanding wastewater surveillance to communities that have been historically marginalized.

Third, working with tribes and regional tribally-designated organizations (such as tribal epidemiology centers) requires an awareness of and respect for tribal governance and historical injustices. Tribes are sovereign nations and tribal resources, relationships with federal and state governments, available staffing, and required legal documentation to engage with surveillance programs may vary greatly by tribe. NWSS has worked closely with tribes on wastewater surveillance activities, ensuring that data ownership and sharing considerations are discussed with the tribe at the outset, and that their needs are addressed in any data use agreements.

Fourth, cohesive partnerships with WWTPs are critical to wastewater surveillance programs. To facilitate these partnerships, wastewater data should be regularly disseminated to WWTP partners, who can use these data to better understand the composition of their incoming wastewater, ensure staff are protected against potential exposures, and document their role in supporting community health. To maintain regular communication with WWTPs, NWSS has partnered with the Water Research Foundation (WEF), a not-for-profit technical and educational organization that represents water quality professionals around the world.

Fifth, regular communication between surveillance system partners is critical to maintaining an operational system. NWSS holds regular meetings with health departments and its commercial contractor and has several online discussion forums. This provides valuable space for knowledge sharing and problem solving among implementing partners.

Sixth, early standardization of wastewater sampling, processing, and testing methods should be prioritized to facilitate data comparisons across sites and time. Similarly, standardizing data structures and submission processes is important to improve data quality and utility. This includes establishing and updating metadata standards for wastewater pathogen genomics. For SARS-CoV-2 sequencing, NWSS established detailed metadata standards early and continues to update these standards to improve transparency, interoperability, and reproducibility of sequencing results. However, standardization of other wastewater methods remains a priority of NWSS.

Seventh, it’s important for wastewater surveillance programs to provide data interpretation and utility guidance to partners. This may include target-specific guidance on how to respond to detections and increases in wastewater concentration levels, detailed information on wastewater metrics, and regularly updated talking points that can be used to consistently respond to public inquiries.

Finally, wastewater data should be used to complement, not replace, data from other surveillance systems. To this end, integrating data from other surveillance systems, including syndromic and case-based surveillance systems, with wastewater data should be a priority. This can be challenging; it requires evaluating population and geographic coverage, temporal resolution, and data timeliness of different surveillance systems and developing a model to accurately combine data from multiple sources. NWSS has integrated COVID-19 case data with wastewater data for sites with known numbers of cases residing within sewershed boundaries, but aligning data across sites and targets from other surveillance systems remains a priority of NWSS.

### 9.4. Future directions

NWSS continues to expand and evolve to meet growing public health needs. First, NWSS will continue to coordinate with STLT partners and its commercial contractor to expand wastewater surveillance to under-represented geographic areas. Initially, NWSS prioritized the enrollment of larger WWTPs to ensure greater population coverage. Now, as NWSS enrolls WWTPs serving smaller communities, the number of individuals represented by NWSS data may not increase meaningfully, but geographic coverage will increase, particularly in rural areas where access to health care may be limited. In areas with limited health care access, wastewater surveillance can provide valuable information for determining disease burden and allocating resources, thus helping to improve health equity.

Second, NWSS will continue to work with partners to add additional targets to wastewater surveillance. As new targets are added, NWSS will refine its recommended sampling strategy, including the number and distribution of sampling sites and frequency of sampling, to ensure sampling is data-driven and tailored to specific targets. As of December 2022, NWSS receives data for SARS-CoV-2 and MPXV. In the future, NWSS will recommend that partners test for additional priority targets, potentially including viral pathogens (e.g., norovirus, adenovirus, respiratory syncytial virus, and influenza), bacterial pathogens (e.g., *Campylobacter jejuni*), parasitic pathogens (e.g., Cyclospora cayetanensis), fungal pathogens (e.g., Candida auris), and antibiotic resistance genes (e.g., colistin, vancomycin, and tetracycline resistance). These priority targets were selected through communications within CDC and between CDC and STLT health departments.

Third, NWSS is developing guidance for partners to implement standardized approaches to wastewater sampling, processing, testing, and data submission. Initially, NWSS sites had the flexibility to use a variety of different methods (Beattie et al., 2022; Dimitrakopoulos et al., 2022; Mogili et al., 2023; Barril et al., 2021; Ahmed et al., 2022; Michael-Kordatou et al., 2020), which helped facilitate the rapid expansion of NWSS. However, by shifting to a more standardized approach to wastewater surveillance, NWSS data will be more readily comparable across sites and time.

Fourth, NWSS is developing an ethical framework for wastewater surveillance by building upon existing guidelines on ethical issues for public health surveillance (WHO guidelines on ethical issues in public health surveillance, n.d.). Wastewater surveillance involves generating and handling human health data, and therefore ethical questions regarding the use of these data must be considered. However, at the time of publishing, formal ethical guidelines on wastewater surveillance are lacking (Doorn, 2022). NWSS’s ethical framework will incorporate previously published work in the wastewater surveillance community and considerations raised by NWSS partners, including privacy and stigmatization. The framework will also work to address ethical considerations around emerging technologies and methods.

Lastly, NWSS is working with health departments and other partners to create a comprehensive database of georeferenced WWTP and sewershed data. This will facilitate the evaluation of NWSS population and geographic coverage and comparisons of wastewater surveillance data to clinical surveillance data.

### 9.5. Conclusions

Through partnering with STLT health departments, NWSS built a robust surveillance platform to track SARS-CoV-2 infections in U.S. wastewater. In less than three years, NWSS nearly quadrupled its population coverage—from 12 % of the U.S. population in 2020 to 45 % in 2022. However, this resulted in an over-representation of larger sampling sites serving more populated communities. As NWSS continues to expand, sites serving rural communities and communities with high SVI are being prioritized for enrollment by NWSS’s commercial contractor and several health departments. To facilitate the rapid expansion of NWSS, NWSS partners have been able to use a variety of wastewater sampling, processing, and testing methods. This has maximized the number of sampling sites that can participate in NWSS, but it has also resulted in large variations in methods, making data comparisons difficult. To address this limitation, NWSS is developing guidance for standardized approaches to wastewater sampling, processing, and testing, which will facilitate data comparisons across sites and time. NWSS continues to expand and improve. Due to its flexible structure, it was a simple process to add MPXV testing in response to the 2022 mpox outbreak. As public health needs change, additional targets may be added to NWSS to respond to emerging and existing health threats. NWSS provides powerful data that are timely and independent of clinical symptoms, healthcare-seeking behaviors, and testing access—NWSS data provide invaluable insights into community infection trends that can be used to help guide public health action.

## Figures and Tables

**Table 1:** Characteristics of CDC’s National Wastewater Surveillance System (NWSS) sampling sites and samples, September 1, 2020 – December 31, 2022^a^.

	September–December 2020	January–December 2021	January–December 2022	December 2022^b^
Characteristics of wastewater sampling sites				
Sites, no.	295	671	1750	1567
Sites actively collecting samples at year’s end, no.^c^	293	621	1567	1567
Millions of persons served by all sites, no. (% of U.S. population)^d^	38 (12)	65 (20)	150 (45)	145 (44)
Thousands of persons served per site, median (IQR)	24 (10, 84)	21 (8, 75)	20 (6, 66)	22 (7, 73)
Site location, no. (%)				
WWTP	287 (97)	617 (92)	1546 (88)	1398 (89)
Upstream of a WWTP	8 (3)	54 (8)	204 (12)	169 (11)
Targets included in testing, no. (%)^e^				
SARS-CoV-2 only	295 (100)	671 (100)	1289 (74)	1107 (71)
SARS-CoV-2 and MPXV	0 (0)	0 (0)	461 (26)	460 (29)
WWTP size in million gallons of wastewater received per day, median (IQR)	8 (3,21)	7 (3,20)	6 (2, 17)	6 (2, 18)
Site management, no. (%)				
Local Health Department	0 (0)	46 (7)	58 (3)	49 (3)
State Health Department	295 (100)	625 (93)	1201 (69)	1072 (68)
Commercial Contractor	0 (0)	0 (0)	394 (23)	349 (22)
Academic Partner Program	0 (0)	0 (0)	97 (6)	97 (6)
Sites located on tribal lands, no. (%)	0 (0)	0 (0)	11 (1)	7 (0)^f^
Average percentage of wastewater from industrial sources received by WWTP, median (IQR)^g^	5 (2,12)	5 (2,13)	5 (1,10)	5 (2, 12)
WWTPs receiving wastewater from a combined sewer system, no. (%)^g,h,i^	34 (26)	70 (34)	159 (44)	149 (44)
Laboratories testing samples, no.	23	58	104	78
No. of samples collected per site per week, median (IQR)	1 (1,2)	1 (1, 2)	2 (1, 2)	2 (1, 2)
Characteristics of wastewater samples				
Samples collected, no.	6062	36,030	111,511	10,965
Time (in days) between sample collection and testing, median (IQR)	2 (1, 4)	2 (1, 4)	2 (1, 4)	2 (1, 4)
Sewage travel time (in hours) from sewage source to sampling site, median (IQR)	9 (5, 15)	9 (5, 15)	6 (3,12)	6 (3, 12)
Sample collection method, no. (%)^h,j^				
Grab	438 (7)	4460 (12)	12,912 (12)	1308 (12)
Manual composite	0 (0)	624 (2)	3210 (3)	296 (3)
Flow-weighted composite	3613 (60)	18,826 (52)	44,665 (40)	3968 (36)
Time-weighted composite	2007 (33)	12,120 (34)	50,243 (45)	5343 (49)
Passive	0 (0)	0 (0)	481 (0)	50 (0)
Sample type^k^				
Untreated wastewater	5770 (95)	31,933 (89)	93,890 (84)	8611 (79)
Wastewater post grit removal	0 (0)	1483 (4)	12,981 (12)	1819 (17)
Primary effluent	0 (0)	0 (0)	90 (0)	15 (0)
Primary sludge	292 (5)	2614 (7)	4550 (4)	520 (5)
Sample volume (in mL), median (IQR)^l^	75 (40, 180)	50 (40, 100)	40 (40, 40)	40 (20, 40)
Targets included in testing, no. (%)^e^				
SARS-CoV-2 only	6062 (100)	36,030 (100)	104,865 (94)	8406 (77)
SARS-CoV-2 and MPXV	0 (0)	0 (0)	6646 (6)	2559 (23)
PCR type^h,m^				
RT quantitative PCR or quantitative PCR	3524 (58)	19,793 (55)	50,236 (45)	4655 (42)
RT digital PCR or digital PCR	0 (0)	592 (2)	19,954 (18)	1983 (18)
RT droplet digital PCR or droplet digital PCR	2538 (42)	15,645 (43)	41,316 (37)	4327 (39)
Endogenous wastewater control				
Pepper Mild Mottle Virus	2866 (47)	15,397 (43)	65,315 (59)	7263 (66)
CrAssphage	877 (14)	4065 (11)	6448 (6)	520 (5)
F+ RNA Coliphage	0 (0)	1 (0)	192 (0)	13 (0)
None	2319 (38)	16,567 (46)	39,556 (35)	3169 (29)

Abbreviations: CDC, Centers for Disease Control and Prevention; NWSS, National Wastewater Surveillance System; no., number; IQR, Interquartile Range; WWTP, Wastewater Treatment Plant; MPXV, monkeypox virus; RT, Reverse Transcription; PCR, Polymerase Chain Reaction; mL, milliliters.

a Sites were included if they collected ≥1 sample during the time period. Samples were included if they were collected during the time period.

b Estimates for December 2022 are shown to summarize sites actively collecting samples at the end of the surveillance summary period.

c Sites that collected samples in December of the respective year were considered to be actively collecting samples at year’s end.

d U.S. Census Bureau population estimate as of April 1, 2020 (331,449,281 persons) were used to calculate percentages of the U.S. population served by NWSS sites. Sampling sites upstream in the wastewater collection network were excluded from population served estimates. Numbers are rounded to the thousand.

e Targets included in testing were determined using data reported to NWSS as of December 31, 2022. No sites tested for MPXV only.

f One tribal community is served by two sites.

g Includes only WWTPs and not sites upstream of WWTPs.

h Percentages were calculated excluding missing observations.

i A combined sewer system is a sewer system that collects both sewage and stormwater.

j Grab samples are from a single moment in time; composite samples are multiple grab samples pooled at a specified frequency over a set period of time; passive samples are collected from gauze or other absorbent material that is left in wastewater for a set period of time (typically several days). Composite samples can be manual (collected manually at different times), time-weighted (collected at preset time intervals using automated equipment), or flow-weighted (collected over regular time intervals with volume proportional to the flow rate using automated equipment).

k Untreated wastewater is wastewater without any form of treatment applied to it. Post grit removal is wastewater after removal of large solids at a treatment plant but prior to a primary clarifier. Primary effluent is effluent from the primary clarifier. Primary sludge is sludge from the primary clarifier.

l Calculations exclude sludge samples.

m RT-PCR is used to quantify SARS-CoV-2 concentrations; PCR is used to quantify MPXV concentrations.

**Table 2:** Characteristics of counties served by CDC’s National Wastewater Surveillance System (NWSS)^a^ compared to all counties in the United States and U.S. territories^b^ by year, September 1, 2020 – December 31, 2022.

	NWSS counties 2020	NWSS counties 2021	NWSS counties 2022	U.S. counties
Number of counties or county-equivalents (%)^c^	211 (7)	410 (13)	976 (30)	3222
County population in thousands, median (IQR)^d^	81 (39, 233)	75 (34, 247)	68 (32,212)	26 (11, 67)
HHS region (headquarters location), no. (%)				
1 (Boston)	3 (1)	3 (1)	47 (5)	67 (2)
2 (New York City)	1 (0)	7 (2)	95 (10)	161 (5)
3 (Philadelphia)	14 (7)	56 (14)	121 (12)	283 (9)
4 (Atlanta)	9 (4)	34 (8)	113 (12)	736 (23)
5 (Chicago)	95 (45)	159 (39)	262 (27)	524 (16)
6 (Dallas)	0 (0)	5 (1)	74 (8)	503 (16)
7 (Kansas City)	45 (21)	70 (17)	99 (10)	412 (13)
8 (Denver)	10 (5)	27 (7)	64 (7)	291 (9)
9 (San Francisco)	11 (5)	19 (5)	47 (5)	96 (3)
10 (Seattle)	23 (11)	30 (7)	54 (6)	149 (5)
RUCC, no. (%)^e^				
1 (most urban)	64 (30)	105 (26)	255 (26)	472 (15)
2	31 (15)	84 (20)	176 (18)	395 (12)
3	28 (13)	58 (14)	138 (14)	369 (11)
4	30 (14)	49 (12)	97 (10)	217 (7)
5	7 (3)	14 (3)	46 (5)	93 (3)
6	32 (15)	48 (12)	118 (12)	597 (19)
7	13 (6)	35 (9)	103 (11)	434 (13)
8	3 (1)	5 (1)	14 (1)	220 (7)
9 (most rural)	3 (1)	12 (3)	29 (3)	425 (13)
SVI, median (IQR)^f^	0.43 (0.21, 0.63)	0.44 (0.23, 0.66)	0.46 (0.26, 0.69)	0.50 (0.25, 0.75)

Abbreviations: NWSS, National Wastewater Surveillance System; IQR, inter-quartile range; HHS, Health and Human Services, no., number; RUCC, Rural-Urban Continuum Codes; SVI, Social Vulnerability Index.

a Counties are considered to be served by NWSS if they were served entirely or partially by one or more NWSS sampling site (i.e., ≥1 site serving the county collected ≥1 sample during the specified timeframe). A site can serve all or part of a sewershed, which is the geographic area contributing wastewater to a sampling location. Sewersheds may cross county or state boundaries.

b U.S. territories include Guam and Puerto Rico.

c County equivalents are geographic regions that are comparable to counties, such as boroughs, parishes, and independent cities. Percentages of all U.S. counties are shown.

d Data are from the U.S. Census Bureau 2020 Decennial Census: https://www.census.gov/programs-surveys/decennial-census/about/rdo/summary-files.html. Estimates are rounded to the thousand.

e Data are from the U.S. Department of Agriculture (USDA) 2013 Urban-Rural Continuum Codes dataset: https://www.ers.usda.gov/data-products/rural-urban-continuum-codes/.

f Data are from the CDC/Agency for Toxic Substances and Disease Registry (ATSDR) 2020 Social Vulnerability Index dataset: https://www.atsdr.cdc.gov/placeandhealth/svi/index.html. SVI is a measure from 0 to 1, with higher values indicating greater vulnerability in a public health emergency.

**Table 3:** CDC’s National Wastewater Surveillance System (NWSS) metrics for displaying SARS-CoV-2 concentration data to NWSS partners on the Data Collation and Integration for Public Health Event Response (DCIPHER) dashboard and to the public on the COVID Data Tracker webpage^a^.

Metric name	Description
15-day rolling detection proportion^b^	Proportion of samples from a site that had SARS-CoV-2 detected over the previous 15 days. SARS-CoV-2 is detected when concentrations are greater than the limit of detection, which is laboratory-specific.
Percent change^b^	Linear change in SARS-CoV-2 concentrations from a site over time (the previous 8 or 15 days) or for a given number of samples (the previous 3 or 5 samples), calculated from the slope of a log-normal linear regression line.
Trends^c^	SARS-CoV-2 concentrations for a site categorized as sustained decrease, decrease, plateau, increase, or sustained increase. Categorizations are determined from the direction and statistical significance of the slope of the “Percent total change” metric regression line. Sustained trends (sustained increase or sustained decrease) are based on the 5 most recent samples. If a significant sustained trend is not found, shorter term trends (increase or decrease) are evaluated based on the 3 most recent samples. If no statistically significant trend is found, the trend is classified as plateau.
Percentiles^b^	Quintiles to compare normalized SARS-CoV-2 wastewater concentrations for a site’s most recent sample to historic samples for that site.

a https://covid.cdc.gov/covid-data-tracker/#wastewater-surveillance

b Displayed on both the DCIPHER dashboard and COVID Data Tracker.

c Displayed on the DCIPHER dashboard only.

## Data Availability

Data are unavailable to access due to concerns about identifiability of sampling sites, however a subset of NWSS data are available publicly on the CDC’s COVID Data Tracker.

## HIGHLIGHTS

- CDC’s National Wastewater Surveillance System (NWSS) launched in September 2020
- Tracks SARS-CoV-2 in U.S. wastewater to complement COVID-19 case surveillance.
- Transformed local wastewater surveillance efforts into a national surveillance system.
- Rapidly expanded to cover ≈47 % of the U.S. population by December 2022.
- Continues to expand and evolve, including having added monkeypox virus testing.

## Footnotes

1 ^§^See e.g., 45 C.F.R. part 46.102(l)(2), 21 C.F.R. part 56; 42 U.S.C. §241(d); 5 U.S.C. §552a; 44 U.S.C. §3501 et seq.

## Abbreviations:

NWSS: National Wastewater Surveillance System
WWTP: Wastewater treatment plant
CDC: Centers for Disease Control and Prevention
STLTs: State, tribal, local, and territorial health departments
EPA: Environmental Protection Agency
ECHO: Enforcement and Compliance History Online
ICIS: Integrated Compliance Information System
FRS: Facility Registry Service
SVI: Social vulnerability index
ELC: Epidemiology and Laboratory Capacity for Prevention and Control of Emerging Infectious Diseases Cooperative Agreement
RUCC: Rural-urban continuum codes
HHS: Health and Human Service
RT-dPCR: Reverse transcription digital polymerase chain reaction
RT-ddPCR: Reverse transcription droplet digital polymerase chain reaction
RT-qPCR: Reverse transcription quantitative polymerase chain reaction
FDA: Food and Drug Administration
NCBI: National Center for Biotechnology Information
SRA: Sequence Read Archive
MPXV: Monkeypox virus
DCIPHER: Data Collation and Integration for Public Health Event Response
WEF: Water Research Foundation
